# Preparation of Fe_3_O_4_/C Composite Material from Red Mud for the Degradation of Acid Orange 7

**DOI:** 10.3390/ma18010151

**Published:** 2025-01-02

**Authors:** Jiaxing Cai, Yunye Cao, Bingfei Yang, Jiajie Li, Michael Hitch

**Affiliations:** 1 School of Materials Science and Chemical Engineering, Ningbo University, Ningbo 315211, China; 2 Engineering Research Center for Silicate Solid Waste Resource Utilization of Hebei Province, Hebei GEO University, Shijiazhuang 050031, China; 3 Key Laboratory of Ministry of Education for Efficient Mining and Safety of Metal Mines, School of Resource and Safety Engineering, University of Science and Technology Beijing, Beijing 100083, China; 4 Faculty of Science, University of the Fraser Valley, Abbotsford, BC V2S 7M8, Canada

**Keywords:** Red mud, Fe_3_O_4_/C composite material, Magnetization roasting, COD

## Abstract

This study presents a novel Fe_3_O_4_/C composite material synthesized from red mud through a process of magnetic roasting and separation. The research explores the impact of Fe_3_O_4_/C dosages, sodium persulfate (PS) concentrations, and initial solution pH on the chemical oxygen demand (COD) removal efficiency using Acid Orange 7 as a model pollutant. Optimal conditions were identified as 3 g/L Fe_3_O_4_/C, 20 mM PS, and an initial pH of 2, achieving a 94.11% COD removal efficiency within 30 min. X-ray diffraction and photoelectron spectroscopy analyses confirmed that the magnetization roasting process effectively transformed red mud’s ferric oxide (Fe_2_O_3_) into magnetite (Fe_3_O_4_). Concurrently, Fe_3_O_4_ interacted with residual carbon to form the Fe_3_O_4_/C composite. This composite demonstrated superior catalytic performance, along with excellent recyclability and reusability.

## 1. Introduction

Red mud (RM) is an alkaline waste produced after the extraction of alumina from bauxite. It contains high quantities of iron [1]. Due to different extraction processes, RM production varies from 1 to 2 tn/tn of alumina [2]. With the increasing alumina production worldwide, red mud accumulation has also increased rapidly. The annual production rate of RM is currently 200 million tonnes approximately, and the cumulative quantities of RM have exceeded 4 billion tonnes [3]. The fine particle size, high alkalinity (pH 10–13), and trace amounts of heavy metals in RM can pose significant threats to both the environment and human health [4]. Research on using RM is ongoing, focusing on extracting valuable metals [5], shaping building materials [6], manufacturing adsorbents [7], etc. However, the comprehensive utilization rate of RM is still low [8]. 

Currently, a variety of wastewater treatment methods are available [9,10]. Advanced oxidation processes, which generate highly reactive free radicals, have proven effective in the practical degradation of a wide range of pollutants [11]. Compared with hydroxyl radical (O •H), sulfate radical $\left({{\mathrm{SO}}_{4}^{•}}^{-}\right)\;$is favored because of its higher oxidation potential ($E^{0}\;$= 2.6 V), more comprehensive pH range, higher reaction rate (106–109 M/s), and higher half-life (t = 30–40 μs) produced by choosing the precursor persulfate (PS) and peroxymonosulfate (PMS) with strong oxidation [12]. PS is the most commonly used precursor, activated by thermal [13], UV [14], and photochemical methods [15]; electrocatalysis [16]; and metal ions [17]. The transition metals method has the distinct advantages of low costs and easy preparation. Iron and iron oxides are transition metals used as catalysts. The reactions are as follows [18,19]:

(1) $$\mathrm{Iron}:\;{\mathrm{Fe}}^{0}+2S_{2}O_{8}^{2-}\to {\mathrm{Fe}}^{2+}+2{\mathrm{SO}}_{4}^{2-}+2\left({{\mathrm{SO}}_{4}^{•}}^{-}\right)$$

(2) SO4•−+H2O→SO42−+O •H+H+

(3) $$\mathrm{Iron}\;\mathrm{oxides}:\;{\mathrm{Fe}}^{2+}+S_{2}O_{8}^{2-}\to {\mathrm{Fe}}^{3+}+{\mathrm{SO}}_{4}^{2-}+\left({{\mathrm{SO}}_{4}^{•}}^{-}\right)$$

(4) $${\mathrm{Fe}}^{2+}+\left({{\mathrm{SO}}_{4}^{•}}^{-}\right)\to {\mathrm{Fe}}^{3+}+{\mathrm{SO}}_{4}^{2-}$$

Ferroferric oxide (Fe_3_O_4_) is widely used as a heterogeneous Fenton catalyst material because of its high Fe^2+^ content, low cost, ecological friendliness, and recyclability. The use of pure Fe_3_O_4_ as a catalyst presents certain drawbacks, including a tendency to aggregate, low catalytic activity, and a low utilization rate. To address these limitations of Fe_3_O_4_ in catalytic applications, researchers have often employed specific modifications to Fe_3_O_4_ to mitigate these issues [20]. From previous studies, most Fe_3_O_4_ samples synthesized by researchers were usually obtained by preparing iron salts and then modified in the next step. These preparation methods were not only complex but also contributed to increased production costs.

Red mud (RM), which is rich in iron, serves as a viable source of iron. In this research, the Fe_3_O_4_/C catalyst was synthesized via a straightforward magnetization roasting technique, utilizing RM as the iron feedstock and activated carbon (AC) as a reductant. Acid Orange 7 (AO7)—a dye extensively employed in the textile, food, and cosmetic sectors—was selected to represent the target pollutant. Due to its genotoxic properties, this can lead to serious environmental problems and therefore requires treatment of wastewater containing AO7 before discharge [21]. The chemical oxygen demand (COD) removal efficiency was used to evaluate the performance of Fe_3_O_4_/C. This study aimed to investigate the activation ability and feasibility of Fe_3_O_4_/C to enhance PS decomposition and further degrade AO7.

## 2. Experiment

### 2.1. Chemicals and Reagents

Red mud containing 27.49 wt% Fe was obtained from Shandong Province with a size of ≤1 mm. Activated carbon was bought from Yuanying New Materials Company (Shijiazhuang, Hebei, China), and its particle size was 8~30 μm. Acid Orange 7 (average Mw~350.32) was from Aladdin (Shanghai, China). Sodium persulfate was from the Rhawn agent (Shanghai, China). Sodium hydroxide was from Macklin (Shanghai, China). Sulfuric acid was analytically pure and purchased from Zhongxing Chemical Reagent (Lanxi, Zhejiang, China). Deionized water (DI) was used throughout the experiment. 

### 2.2. Preparation Process of Fe_3_O_4_/C

The synthesis of Fe_3_O_4_/C composite material was as follows: 10 g RM was thoroughly mixed with 1 g activated carbon (AC) and then poured into a clay–graphite crucible and compacted with a grinding rod. To maintain the reducing atmosphere, a lid was placed on the top of the clay–graphite crucible. The whole roasting experiment was carried out in a muffle furnace. When the furnace reached 800 °C, the clay–graphite crucible containing the sample was placed into the muffle furnace for 45 min. After the roasting process, the crucible was removed from the furnace, and the sample was poured into water for cooling. Afterwards, the sample was wet ground at a speed of 205 r/min and separated via magnetic separation (CXG-008SD, 100 mT, Beijing, China). The sample was collected and vacuum-dried in an oven at 60 °C for 12 h.

### 2.3. Characterization of Fe_3_O_4_/C

To characterize the Fe_3_O_4_/C composite material, the morphology of the Fe_3_O_4_/C was analyzed via scanning electron microscopy (SEM, VEGA3, Tescan, Brno, Czech Republic), and the elements of the synthesized products were analyzed using an energy dispersive spectrometer (EDS, NORAN System 7, Thermo Scientific, Waltham, MA, USA). An X-ray diffraction pattern analyzer obtained the catalyst’s crystal orientation with a Cu-Kα source, and the catalyst was scanned in the 2θ range of 10–90° with a step size of 0.1°(XRD, Bruker D8 Advance, Karlsruhe, Germany). The material’s elemental composition was obtained using X-ray photoelectron spectroscopy (XPS, Thermo Scientific ESCALAB 250Xi, Waltham, MA, USA).

### 2.4. Degradation Experiment 

The details of the entire degradation experiment were as follows: First, the COD value of the 50 mg/L homogeneous solution was prepared by mixing 10 mg of AO7 with 200 mL of DI in a 250 mL beaker. Then, a certain amount of PS (5–25 mM) was added to the beaker. Finally, Fe_3_O_4_/C (0–4 g/L) was added to the beaker and stirred in a water bath (HJ-4S, Shanghai, China) at 350 ± 20 rmp. Then, 5 mL of the supernatant at different reaction time points was collected and passed through a 0.45 μm filter. The initial pH of the solution was determined using a pH meter (pH 848, Dongguan, China) and adjusted using H_2_SO_4_ (1:3) and NaOH (1 M). The effects of Fe_3_O_4_/C dosage concentrations and the initial pH of the solution (PH = 2, 3, 6.4, 8) on AO7 degradation were investigated.

### 2.5. Analytical Methods 

The rapid digestion method was used to determine COD [22], and a spectrophotometer (JC-N, Juchuang Environmental, Qingdao, China) was used to characterize COD’s organic degradation efficiency. The COD degradation efficiency was defined as follows:

(5) $$D=\frac{{[\mathrm{COD}}_{0}]\;{-\;[\mathrm{COD}}_{t}]}{\left[{\mathrm{COD}}_{0}\right]}\;\times \;100\%$$

where${\;[\mathrm{COD}}_{0}]\;$and ${[\mathrm{COD}}_{t}]$ (mg/L) represent the initial COD concentration and the COD concentration at time t (min) of the AO7 solution, respectively.

## 3. Results and Discussion

### 3.1. Characterization

**Figure 1:**
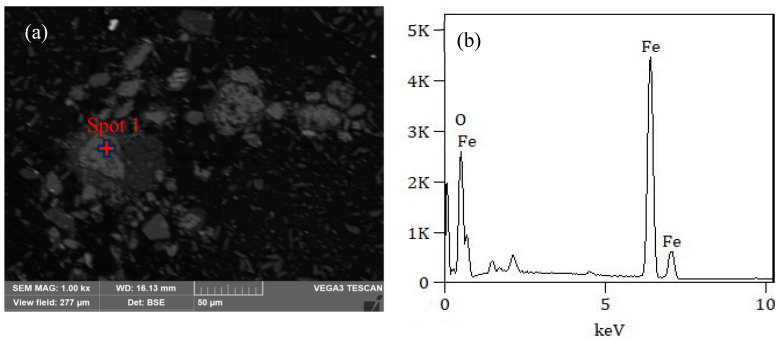
(**a**) SEM image of the synthesized product; (**b**) EDS analysis result of spot 1.

Fe_3_O_4_/C composite materials were synthesized via simple magnetization roasting. Figure 1a shows an SEM image of the synthesized product.

The bright areas in the image represented metal oxides. According to the EDS element analysis of the bright region, the main components were Fe and O. SEM could observe that the shape of the synthesized product was irregular. XRD and XPS further characterized the synthesized products to determine the material properties better.

**Figure 2:**
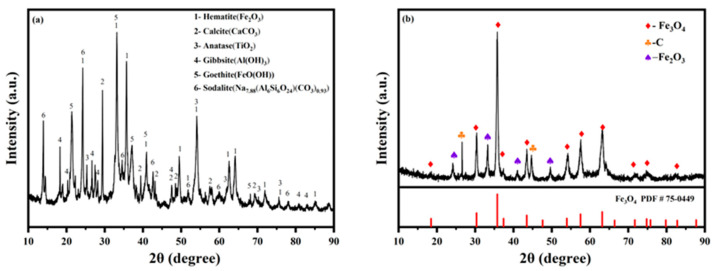
The XRD patterns of (**a**) red mud and (**b**) synthesized products.

The substance was analyzed before and after the reaction, as shown in Figure 2. The qualitative analysis of the red mud was carried out. As shown in Figure 2a, the iron elements in the red mud mainly existed in the form of hematite (Fe_2_O_3_) and goethite (FeO(OH)). The other mineral groups were calcite, anatase, gibbsite, sodalite, etc. The synthesized substances were analyzed, and the results are shown in Figure 2b. It could be observed that the synthesized product had strong diffraction peaks at 2θ = 30.5°, 35.8°, 43.4°, 53.9°, 57.5°, and 63.2°, corresponding to the (2 2 0), (3 1 1), (4 4 0), (4 2 2), (5 1 1), and (4 0 0) planes, respectively. The position and relative intensity of characteristic diffraction peaks with a standard card (Fe_3_O_4_: PDF 75-0049) could be well matched, indicating that the synthetic substance was mainly Fe_3_O_4_.

The primary iron source in red mud was Fe_2_O_3_, and Fe_3_O_4_ was produced via magnetization roasting. The reaction equation in the process was the same as in the literature [23].

The diffraction peaks at 2θ = 26.58° and 44.67° were the characteristic carbon peaks (C: PDF 26-1076). The results showed that the Fe_3_O_4_/C material was successfully prepared by combining the generated Fe_3_O_4_ with excess carbon during the reaction with unreacted Fe_2_O_3_.

**Figure 3:**
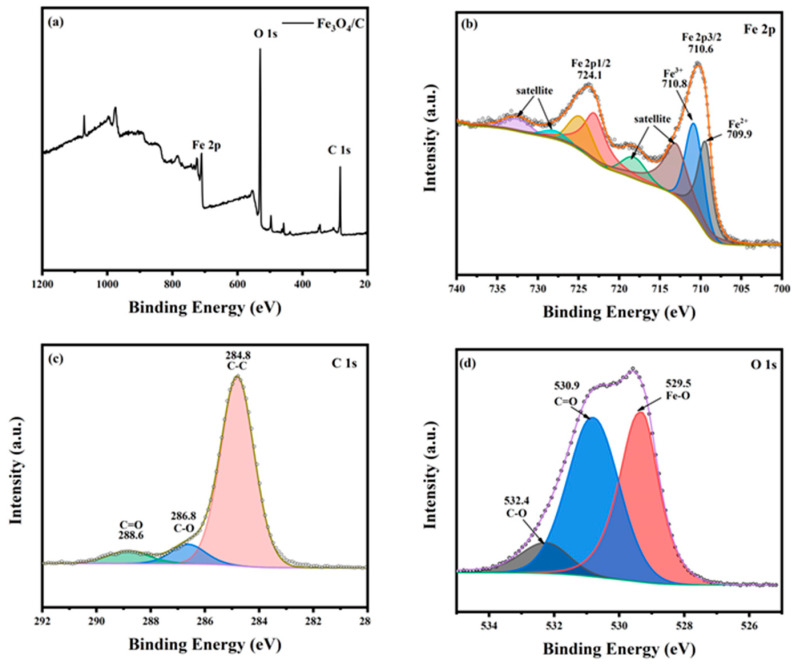
High-resolution XPS spectra of (**a**) Fe_3_O_4_/C, (**b**) Fe 2p, (**c**) C 1s, and (**d**) O 1s.

XPS analysis was further carried out to better understand the sample’s composition, and the results are shown in Figure 3. The chemical composition analysis results of the sample’s surface are shown in Figure 3a. The main components of the sample included Fe, O, and C elements. Furthermore, to obtain more bonding information on the sample’s surface, the fine scanning of Fe 2P spectra and fitting analysis were performed for different valence values of iron ions. The result is shown in Figure 3b, where the peak values of Fe 2p3/2 and Fe 2p1/2 are around 710.6 eV and 724.1 eV, respectively, close to those in references [24,25]. The fitting peaks at 709.9 and 710.8 eV were Fe^2+^ and Fe^3+^ for the octahedral bodies, respectively [26,27]. It was again shown that part of Fe_2_O_3_ in the red mud was converted to Fe_3_O_4_.

The detection of carbon on the sample’s surface via XPS indicated that the Fe_3_O_4_/C composite material had been successfully synthesized. According to the high-resolution C 1s XPS spectrum of Fe_3_O_4_/C, three individual component peaks could be obtained via fitting: binding energy of 284.8 eV, 286.8 eV, and 288.6eV. The causes were attributed to C–C, C–O, and C=O bonds, respectively [25,28]. According to the high-resolution O 1s XPS spectrum of Fe_3_O_4_/C, three individual component peaks could be obtained via fitting: binding energy of 529.5, 530.9, and 532.4 eV. The causes were attributed to the Fe–O, C=O, and C–O bonds [29].

The above results indicated that the main component of the synthesized product was Fe_3_O_4_/C. The Fe^2+^ in the structure could be used as a catalyst to activate the degradation of organic compounds.

### 3.2. Effect of Fe_3_O_4_/C Dosage on Degradation 

**Figure 4:**
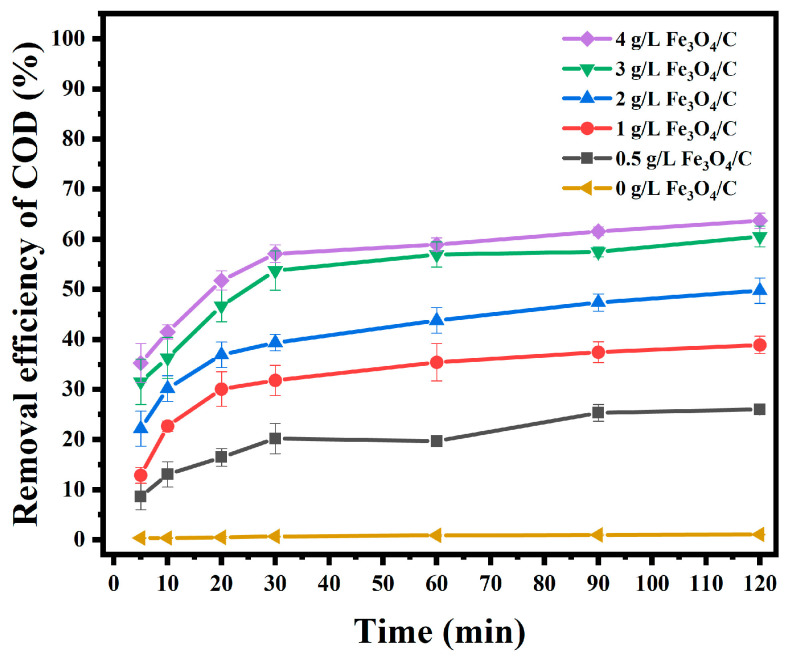
Effect of Fe_3_O_4_/C dosage on degradation.

To investigate the catalytic activity of Fe_3_O_4_/C material on PS, a set of experiments was carried out on the degradation of AO7. The COD removal efficiency was used to evaluate the catalyst’s performance. A set of experiments was designed to assess the COD removal efficiency. The dosage of Fe_3_O_4_/C composite materials ranged from 0 g/L to 4 g/L. Other reaction conditions remained the same: the initial pH = 2, and the concentration of Ps was 5 mM. To reduce the effect of temperature, the reaction temperature was 25 °C. The subsequent reaction temperature remained unchanged. The reaction results are shown in Figure 4.

The experimental results showed that AO7 was almost challenging to degrade when no Fe_3_O_4_/C composites were added, and the COD removal efficiency was only 1.05% after 120 min. As the dosage of Fe_3_O_4_/C composites gradually increased from 0.5 g/L to 4 g/L, the COD removal efficiency gradually increased. This is attributed to the fact that as the amount of Fe_3_O_4_/C gradually increased, more reaction sites were provided, thus facilitating the degradation of AO7. When the dosage of Fe_3_O_4_/C composites was increased to 3 g/L, the COD removal efficiency in 30 min reached 53.75%. When the dosage of Fe_3_O_4_/C composites was further increased, the COD removal efficiency was not significantly increased. With the increase in the dosage of Fe_3_O_4_/C, Fe^2+^ may have combined with the generated ${{\mathrm{SO}}_{4}^{•}}^{-}$ to accelerate the quenching of ${{\mathrm{SO}}_{4}^{•}}^{-}$when more Fe^2+^ was released [30]. The overall reaction trend showed that the COD removal efficiency was the most rapid in the first 30 min, and the degradation efficiency slowed down when the reaction exceeded 30 min.

Considering COD removal efficiency and economic costs, the optimal dosage of Fe_3_O_4_/C composite material was 3 g/L. This dosage was used in subsequent tests.

### 3.3. Effect of PS Concentration on Degradation

**Figure 5:**
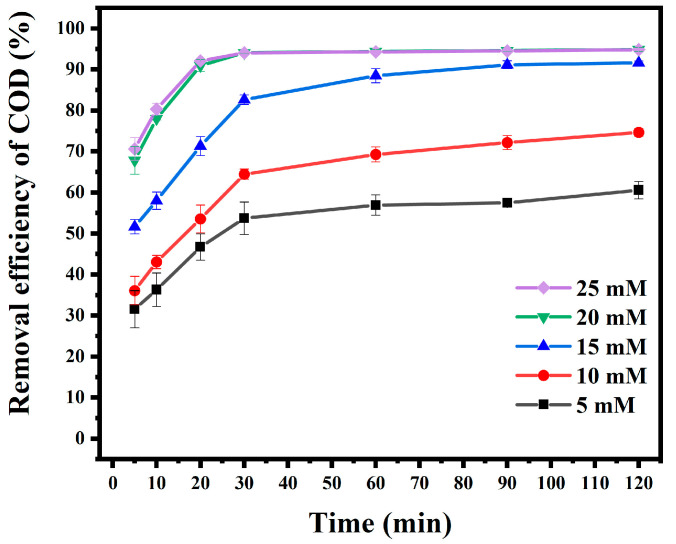
Effect of PS concentration on degradation.

The concentration of PS has a decisive effect on the degradation of AO7. A set of PS concentrations ranging from 5 to 25 mM was designed to investigate this relationship. The reaction’s initial pH was 2; the experimental result is shown in Figure 5.

According to the experimental results, the COD removal efficiency increased with an increase in PS concentration, and there was a direct relationship between them. When the concentration of PS was low, it was not enough to completely degrade AO7. Increasing the concentration of PS could release more ${{\mathrm{SO}}_{4}^{•}}^{-}$ in the reaction system. This significantly improved the COD removal efficiency. When the concentration of PS was increased to 20 mM, the COD removal efficiency at 30 min could reach 94.11%. When the concentration of PS was further increased, the COD removal efficiency was only 30 min before the degradation of PS was increased. When the concentration of PS exceeded 20 mM, there was no significant increase in the COD removal efficiency. Under the fixed dosage of Fe_3_O_4_/C, the COD degradation efficiency increased with the rise in PS concentrations. Until the two reached a particular proportion, the further increase in PS concentration did not significantly improve degradation efficiency. Therefore, according to the experimental results, the degradation of AO7 was best when the PS concentration was 20 mM. Subsequent experiments used this condition.

### 3.4. Effect of Initial pH of Reaction Solution on Degradation

**Figure 6:**
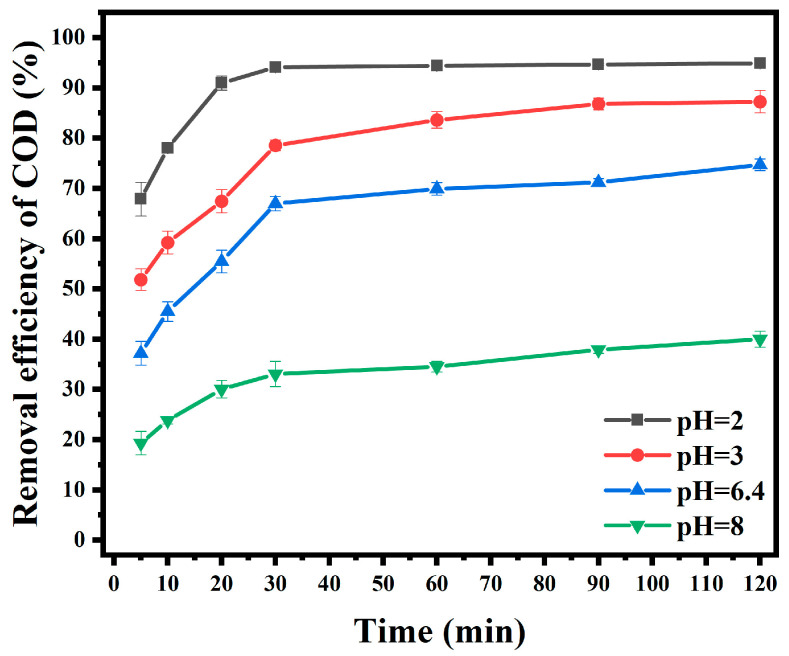
Effect of the initial pH of solution on degradation.

To explore the effect of the initial pH of solution on AO7 degradation, different initial pH (pH = 2, 3, 6.4, 8) values were investigated, and the experimental results are shown in Figure 6.

According to the analysis of the experimental results, the COD removal efficiency decreased gradually with the increase in pH. The reason may be that an increase in the initial pH value will produce iron precipitation, resulting in lower degradation efficiency [31]. At the same time, it was found that the AO7 solution could still degrade under natural conditions (pH = 6.4), and the COD removal efficiency could reach 66.97% at 30 min. This could be more suitable for sewage treatment in real life. A comparison of Fe_3_O_4_/C with other already reported catalysts is given in Table 1.

### 3.5. Stability and Reusability of Fe_3_O_4_/C

**Figure 7:**
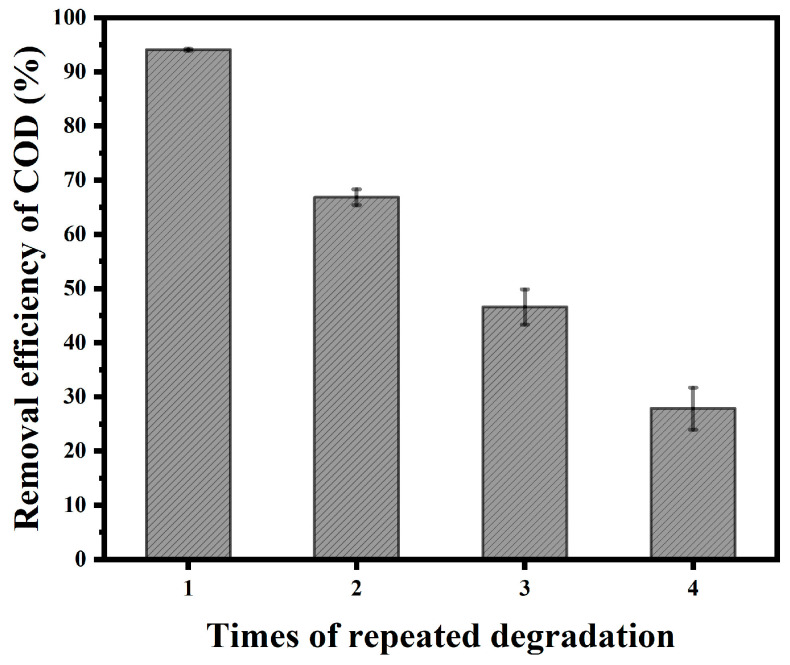
Effect of repetition times on degradation.

To explore the sustainability and recyclability of Fe_3_O_4_/C composite materials under the best conditions (the dosage of Fe_3_O_4_/C was 3 g/L, the concentration of PS was 20 mM, and the initial pH of the solution was 2), the COD removal efficiency after different reaction times at 30 min was investigated, and the experimental result is shown in Figure 7.

According to the experimental results, the degradation efficiency of AO7 dramatically decreases with an increase in the repetition times of Fe_3_O_4_/C composite materials. The COD removal efficiency after repeated use at 2, 3, and 4 times at the 30 min point was 66.89%, 46.63%, and 27.84%, respectively. The reason was that after repeated many times, the Fe^2+^ content contained in the sample was significantly reduced, thus affecting the degradation of AO7.

Preparing Fe_3_O_4_/C from red mud not only solves the environmental pollution problem of red mud but also transforms it into a valuable resource. The Fe_3_O_4_/C/PS system could effectively remove organic pollutants in wastewater. It also realized the recycling of resources and the environmental protection of red mud, and it promoted the comprehensive utilization of red mud for sustainable development.

## 4. Conclusions

In this research, red mud was utilized to synthesize the Fe_3_O_4_/C composite material through a magnetization roasting process. The synthesized sample was characterized using X-ray diffraction (XRD) and X-ray photoelectron spectroscopy (XPS), confirming the successful preparation of the composite. The study also examined the impact of various reaction conditions on the efficiency of chemical oxygen demand (COD) removal. The optimal conditions for degrading Acid Orange 7 (AO7) were determined to be an Fe_3_O_4_/C dosage of 3 g/L, a sodium persulfate (PS) concentration of 20 mM, and an initial solution pH of 2. With these parameters, the COD removal efficiency achieved 94.11% within 30 min. The findings indicated that the AO7 solution was effectively degraded under natural conditions. Moreover, the Fe_3_O_4_/C composite material not only demonstrated superior catalytic performance but also showed potential for efficient recycling and reuse.

## Figures and Tables

**Table 1:** Comparison of Fe_3_O_4_/C with other already reported catalysts.

Types of Catalysts	Target Effluent	Pollutant Concentration	Removal Rate (%)	References
Fe_3_O_4_/MC	Tetracycline	50 mg/L	92.9	[20]
Fe_3_O_4_@PS@Ag	4-nitrophenol	3 mM	>99	[32]
Fe_3_O_4_@TiO_2_	Thallium	8.5 mg/L	94.7	[33]
Fe_3_O_4_@C/CDs-Ag	Penicillin	100 μM	>97	[34]
Fe@Fe_3_O_4_-C	Rhodamine B	50 mg/L	100	[35]
ZnO@Fe_3_O_4_	AO7	7 mg/L	80	[36]
Fe_3_O_4_-C@MoS_2_	Tetracycline	20 mg/L	>97	[37]
Fe_3_O_4_/C	AO7	50 mg/L	94.11	This work

## Data Availability

Data is contained within the article. Further inquiries can be directed to the corresponding author.
